# Inclusion Body Bead Size in *E. coli* Controlled by Physiological Feeding

**DOI:** 10.3390/microorganisms6040116

**Published:** 2018-11-25

**Authors:** Julian Kopp, Christoph Slouka, Daniel Strohmer, Julian Kager, Oliver Spadiut, Christoph Herwig

**Affiliations:** 1 Christian Doppler Laboratory for Mechanistic and Physiological Methods for Improved Bioprocesses, Institute of Chemical Engineering, Vienna University of Technology, 1060 Vienna, Austria; julian.kopp@tuwien.ac.at (J.K.); daniel.strohmer@gmx.net (D.S.); christoph.herwig@tuwien.ac.at (C.H.); 2 Research Division Biochemical Engineering, Institute of Chemical Engineering, Vienna University of Technology, 1060 Vienna, Austria; julian.kager@tuwien.ac.at (J.K.); oliver.spadiut@tuwien.ac.at (O.S.)

**Keywords:** *E. coli*, recombinant proteins, bioprocess engineering, process control, inclusion body, size

## Abstract

The Gram-negative bacterium *E. coli* is the host of choice for producing a multitude of recombinant proteins relevant in the pharmaceutical industry. Generally, cultivation is easy, media are cheap, and a high product titer can be obtained. However, harsh induction procedures combined with the usage of IPTG (isopropyl β-d-1 thiogalactopyranoside) as an inducer are often believed to cause stress reactions, leading to intracellular protein aggregates, which are so known as so-called inclusion bodies (IBs). Downstream applications in bacterial processes cause the bottleneck in overall process performance, as bacteria lack many post-translational modifications, resulting in time and cost-intensive approaches. Especially purification of inclusion bodies is notoriously known for its long processing times and low yields. In this contribution, we present screening strategies for determination of **i**nclusion body bead size in an *E. coli*-based bioprocess producing exclusively inclusion bodies. Size can be seen as a critical quality attribute (CQA), as changes in inclusion body behavior have a major effect on subsequent downstream processing. A model-based approach was used, aiming to trigger a distinct inclusion body size: Physiological feeding control, using q_s,C_ as a critical process parameter, has a high impact on inclusion body size and could be modelled using a hyperbolic saturation mechanism calculated in form of a cumulated substrate uptake rate. Within this model, the sugar uptake rate of the cells, in the form of the cumulated sugar uptake-value, was simulated and considered being a key performance indicator for determination of the desired size. We want to highlight that the usage of the mentioned screening strategy in combination with a model-based approach will allow tuning of the process towards a certain inclusion body size using a q_s_ based control only. Optimized inclusion body size at the time-point of harvest should stabilize downstream processing and, therefore, increase the overall time-space yield. Furthermore, production of distinct inclusion body size may be interesting for application as a biocatalyst and nanoparticulate material.

## 1. Introduction

The gram-negative bacterium *E. coli* might be one of the best-characterized organisms and has found its place in many different industrial applications [1,2], because *E. coli* shows very fast replication rates [3,4] on comparatively cheap media [5]. However, the missing glycosylation pattern [1,6,7], as well as intracellular protein production, implies major drawbacks when compared to mammalian cells and yeasts as hosts [8]. Still, proteins that do not need major posttranslational modifications can be produced in bacteria at low costs in short process times [7]. The strain BL21(DE3) created by F. Studier and B. Moffatt back in 1986 [9] is often used on an industrial scale because of very low acetate formation, high replication rates and high production as an effect of the integrated T7-polymerase [9,10,11,12,13,14]. Repressing the lac operon with isopropyl β-d-1 thiogalactopyranoside (IPTG) is still state of the art in most industrial applications [15,16], as the pET-expression-system is known for high replication rates, leading to high amounts of the desired protein [3,12,16].

Inclusion bodies (IB) have emerged from their role as waste products [17] and even are purposely produced in many processes from the 1970s and 80s (e.g., interleukins, insulin) to nowadays [18]. IBs have been believed to be a result of diverse stress reactions, resulting in biologically inactive protein [18,19,20]. Their formation is generally based upon a self-aggregation and overwhelming of the refolding apparatus. Using strong promotors with high production of recombinant proteins, IBs are hard to prevent, but on the other hand side IBs also open up the door for the production of toxic proteins [21]. Intracellular stress can be caused by high temperatures, pH-shifts or due to high feeding rates [22]. However, some of these stress reactions tend to impact in higher yields of product [1]. Still, down-stream processing (DSP) and especially the refolding unit operation is the most time-consuming step in gaining the correctly folded product from *E. coli* cultivations [17,18,19,20], which requires significantly more technology and time, when purifying protein aggregates for pharmaceutical products [19,23]. In recent years IBs were found to exhibit not only inactive protein structures, but also parts which show enzyme activity [24]. These IBs are nowadays called non-classical Inclusion Bodies (ncIBs) and inherit highly active protein, directly without time-consuming refolding steps [25,26,27]. Active parts of the IB may also be directly used as biocatalysts in different reactions, combining the well mechanical properties with enzymatic reactivity. It has also been shown, that the size has an influence on the biological activity of the protein [28,29]. Beside their enzymatic usage, IBs may also be used based on their mechanical properties as they generally inhibit a distinct bead size combined with high mechanical stability. Therefore, application as nanoparticulate material and as material for tissue engineering is considered [17,28,30]. For all these mentioned applications, IB bead size can be regarded as critical quality attribute (CQA). Changes in size are known to be based on the harvest time during the cultivation. In a recent study, we analyzed detailed effects of classical process parameters pH and temperature on the IB bead size as a function of the induction time [22]. Therefore, control of IB size during the cultivation is an important issue as size is a CQA during these processes. Process performance needs to be directed towards optimal size needed in DSP for pharmaceutical relevant components.

Effects of feeding strategies onto product quality have been already investigated by Spadiut et al. [31] as alterations of the specific growth rate are known to have major influences onto product formation. As high recombinant protein production does implement changes in cell physiology, it is known that there is a decreased growth rate over the duration of induction time [32]. Also biomass formation does suffer from protein induction as it has been referred that biomass yields decrease over induction time, especially when compared to mock strains [33]. Helping to optimize feeding strategies, the term q_s_ has been introduced, determining specific sugar uptake rates, per gram biomass in certain time intervals [31]. Within this study, it was also shown that a stepwise increase, such as a q_s_-ramp, is superior to a constant feeding profile in *Pichia pastoris* protein production. The influence of q_s_ ramps onto inclusion body formation was performed by Reichelt et al. [34]. Within this study also the term “q_s,crit_” was determined, describing the maximum physiological substrate uptake rate. It was shown within this study, that the physiological feeding rate in *E. coli* is highly dependent onto process parameters and consists of a rather “dynamic nature”. Physiological capacities decline during induced bioprocesses leading to substrate accumulation [35].

In this study, we followed the hypothesis that the specific substrate uptake rate may be a suitable process parameter to steer IB CQA, such as IB size. We performed cultivations with a *E. coli* BL21(DE3) strain, producing a recombinant pharmaceutical protein coupled to a N-pro-fusion protein [36], expressed exclusively as an IB with the goal to control inclusion body size within the cells based on physiological feeding. Optimized process parameters for temperature (T) and pH were used throughout the whole work, derived in from a different previous study. The specific substrate feeding rate (q_s,C_) was adapted using glycerol and glucose and responses onto IB bead size were analyzed. Usage of glycerol as media is comparable cheap and since glycerol is regarded as a waste product [37] it might find further applications in future manufacturing. The degree of reduction on glycerol is slightly higher when compared to glucose and titer based productivity seemed to be positively influenced using glycerol as a primary carbon source [38]. We show that IB bead size can be adjusted via the amount of fed carbon source, when keeping T and pH constant during induction. Size variations could be detected throughout the cultivations using scanning electron microscopy. The knowledge generated by these cultivations is used to model IB size based on the glycerol consumption and predict IB size of a cultivation in real time. Using this model-based approach, a defined IB-size at the time point of harvest can be simulated, which should enhance performance as biomaterials or lead to optimized DSP for pharmaceutical products.

## 2. Materials and Methods

### 2.1. Bioreactor Cultivations

All cultivations were carried out with the strain *E. coli* BL21(DE3) consisting of the pet30a plasmid system. The target protein strain was linked to a N-pro fusion protein, which is only expressed as IB (no soluble form) [36]. All bioreactor and preculture cultivations were carried out using a defined minimal medium referred to DeLisa et al. (1999) [5]. Batch media and the preculture media had the same composition with different amounts of glycerol and glucose as carbon source (C-source) respectively. The C-source concentrations for the phases were following.

As pET30a has a Kanamycin resistance gene, antibiotic was added throughout all fermentations, in a final concentration of 0.02 g/L. Precultures were performed using 500 mL high yield flasks (containing the C-source concentrations given in Table 1 or 100 mL Erlenmeyer shake flasks for DasGIP systems). They were inoculated with 1.5 mL of bacteria solution stored in cryo stocks at −80 °C and subsequently cultivated for 20 h at 230 rpm in an Infors HR Multitron shaker (Infors, Bottmingen Switzerland) at 37 °C.

All cultivations were either performed in a Sartorius Biostat Cplus bioreactor (Sartorius, Göttingen, Germany) with 10 L working volume or in a DASGIP Bioreactor 4-parallel fermenter system (max. working V.: 1.7 L; Eppendorf, Hamburg, Germany). Cultivation offgas was analyzed by gas sensors-IR for CO_2_ and ZrO_2_ based for O_2_ (Blue Sens Gas analytics, Herten, Germany). The cultivations were controlled using Lucullus process control system (SecureCell, Schlieren, Switzerland) or the provided DAS-GIP-control system, DASware-control, which logged the process parameters. The reactors were continuously stirred at 1400 rpm.

During induction phase, pH was kept constant at 6.7 and temperature at 31.5 °C and controlled with base only (12.5% NH_4_OH), while acid (10% H_3_PO_4_) was added manually, if necessary. The pH was monitored using a pH-sensor EasyFerm Plus (Hamilton, Reno, NV, USA). Aeration was carried out using mixture of pressurized air and pure oxygen at 2 vvm. Oxygen was added accordingly to keep dissolved oxygen (dO_2_) always higher than 30%. The dissolved oxygen was monitored using a fluorescence dissolved oxygen electrode Visiferm DO (Hamilton, Reno, NV, USA).

### 2.2. Cultivation Scheme and q_s_ Adaption

Inoculation was always done with one tenth of the batch media volume. Preculture showed an OD_600_ of approximately 7 after cultivation. The batch process, performed at 37 °C took around 6–7 h and was finished, visible by a drop in the CO_2_-signal. The 20 g/L of C-source usually resulted in a biomass of 9–10 g/L. After the batch was finished a non-induced fed-batch was started overnight, at 35 °C and adapting the q_s,C_, based on the biomass after the batch, in an exponential feed value to gain a biomass of approximately 30 g/L. The non-induced fed-batch generally tool about 16 h with a q_s,C_ value of about 0.2 g/g/h. Afterwards q_s,C_ was adapted to a certain point of interest, temperature was decreased to 31.5 °C and pH 6.7 and stabilized for 30 min before the inducer was added. Induction was always performed with a 0.5 mM IPTG and lasted for highest of 12 h.

For screening of IB bead size static feed-forward q_s_-controls were performed during induction phase [3,39]. Exponential feed was established according to Equation (1), an exponential feed-forward approach to keep q_s,C_ constant [3,39,40,41]:

(1) $$F\left(t\right)=\frac{q_{s,C}*X\left(t\right)*\rho_{f}}{c_{f}}$$

With F being the feedrate [g/h], q*_s,C_* the specific glycerol uptake rate [g/g/h], X(t) the absolute biomass [g], *ρ*_F_ the feed density [g/L] and c_f_ the feed concentration [g/L] respectively. Real q_s,C_ values for the cultivations were calculated after the run, based on DCW values and gravimetrical feed signals.

### 2.3. Analytics

#### 2.3.1. Process Analytics

Samples were always taken after inoculation, upon end of the batch-phase and after the non-induced-fed batch was finished. During the induction period samples were either taken in 60 or 120 min intervals. Generally, biomass was measured using optical density (OD_600_) and dry cell weight (DCW). OD_600_ was measured using a Genesys 20 photometer (Thermo Scientific, Waltham, MA, USA). Since the linear range of the used photometer is between 0.1 and 0.8, samples were diluted with dH_2_O to stay within that range. The dry cell weight was determined by vortexing the sample, pipetting 1 mL of sample solution in a pre-tared 2 mL Eppendorf-Safe-Lock Tube (Eppendorf, Hamburg, Germany) and centrifuged for 10 min at 10,000 rpm at 4 °C. After centrifugation, the supernatant was used immediately for at-line HPLC measurement (see beneath), while the pellet was resuspended with 1 mL of 0.9% NaCl solution and centrifuged at the same conditions. Afterwards, the pellet was dried for at least 48 h at 105 °C.

Glycerol and glucose concentrations were measured via HPLC-method (Thermo Scientific, Waltham, MA, USA) using a Supelcogel-column; Eluent: 0.1% H_3_PO_4_; Flow: 0.5 mL/min or an Aminex HPLC column (Biorad, Hercules, CA, USA) on an Agilent 1100 System (Agilent Systems, Santa Clara, CA, USA) with 4 mM H_2_SO_4_ as running buffer at 0.6 mL/min. Using this method sugar accumulation could be detected as an indication for cell death during our cultivations. Standards had a concentration of 1 to 50 g/L glycerol and glucose respectively The HPLC run lasted always for 30 min and Chromatograms were analyzed using a Chromeleon Software (Dionex, Sunnyvale, CA, USA).

#### 2.3.2. Product Analytics

##### IB Preparation:

5 mL fermentation broth samples were centrifuged at 4800 rpm at 4 °C. The supernatant is discarded and the pellet is resuspended to a DCW of about 4 g/L in lysis buffer (100 mM Tris, 10 mM EDTA at pH = 7.4). Afterwards, the sample was homogenized using a high-pressure homogenizer at 1500 bar for 10 passages (EmulsiflexC3; Avestin, Ottawa, ON, Canada). After centrifugation at 10,000 rpm and 4 °C the supernatant was discarded and the resulting IB pellet was washed twice with ultrapure water and aliquoted into pellets à 2 mL broth, centrifuged (14,000 rpm, 10 min 4 °C) and stored at −20 °C. Buffer washed samples (buffer A: 50 mM Tris, 0.5 M NaCl, 0.02% Tween 80; buffer B: 50 mM Tris, 5 mM EDTA) did not show differences in IB bead size, and only slight differences in purity pattern (compare to [22]).

##### IB Bead Size:

Washed and aliquoted IB samples were resuspended in ultrapure water. 100 µL of appropriate dilution of the suspension were pipetted on a gold-sputtered (10–50 nm) polycarbonate filter (Millipore-Merck, Darmstadt, Germany) using reusable syringe filter holders with a diameter of 13 mm (Sartorius, Göttingen, Germany). 100 µL of ultrapure water were added and pressurized air was used for subsequent filtration. Additional 200 µL of ultrapure water were used for washing. The wet filters were fixed on a SEM sample holder using graphite adhesive tape and subsequently sputtered with gold to increase the contrast of the sample. SEM was performed using a QUANTA FEI SEM (Thermo Fisher, Waltham, MA, USA) with a secondary electron detector [15]. The acceleration voltage of the electron beam was set between 3 to 5 kV. To determine the diameter of the IBs, 50 IBs on SEM pictures were measured using the ImageJ plugin Fiji (Laboratory for Optical and Computational Instrumentation (LOCI), University of Wisconsin-Madison, USA).

##### IB Titer:

For titer measurements, IB pellets were solubilized using solubilization buffer (7.5 M Guanidine Hydrochloride, 62 mM Tris at pH = 8). The filtered samples are quantified by HPLC analysis (UltiMate 3000; Thermo Fisher, Waltham, MA, USA) using a reversed phase column (EC 150/4.6 Nucleosil 300-5 C8; Macherey-Nagel, Düren, Germany). The product was quantified with an UV detector (Thermo Fisher, Waltham, MA, USA) at 214 nm using Novartis BVS Ref. 02 as standard. Mobile phase was composed of acetonitrile and water both supplemented with 0.1% (*v*/*v*) tetrafluoride acetic acid. A linear gradient from 30% (*v*/*v*) acetonitrile to 100% acetonitrile (ACN) was applied. A steep linear gradient from 10% ACN to 30 % ACN in 60 s was followed by a long linear gradient from 30% to 55% and by 3 regeneration steps.

## 3. Results

Within this study, we investigated size dependence based on physiological feeding in *E. coli* during cultivation. The time-dependent harvested and washed IBs were analyzed via SEM and diameter was measured subsequently. A correlation of the IB size based on the fed substrate could clearly be indicated. A hyperbolic relation of the size could be used to establish model-based approach to predict IB size for our model protein during cultivation.

### 3.1. Static q_s,C_ Feed-Forwards Feeding for Size Determination

**Figure 1:**
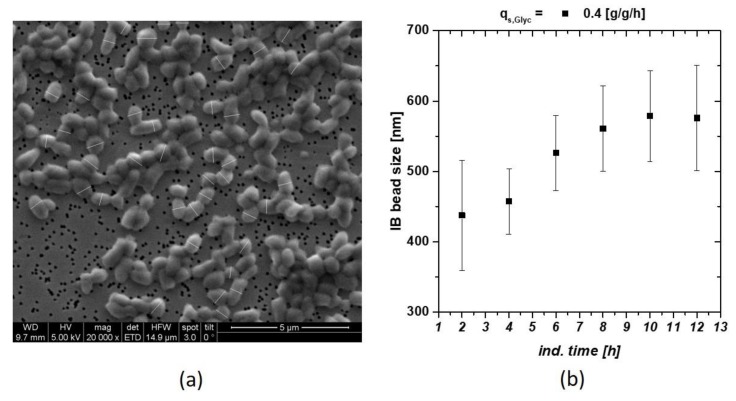
(**a**) Inclusion body (IB) beads at time point of harvest (12 h of induction) including measurement bars (white) after homogenization and subsequent washing with ultrapure water; (**b**) IB bead size at q_s,C_ of 0.4 g/g/h using glycerol as C-source. Late degradation is based on reduction in the viable cell concentration.

Within a screening approach, we tried to trigger the optimal feeding parameter for IB production in *E. coli* processes. Set-point alteration for the specific glycerol or glucose uptake rate q_s,C_, did influence the IB bead size respectively. Classical and physiological process parameters using glucose as carbon source had already been analyzed in recent work [22]. These findings resulted in the optimized pH and temperature parameters used in this study (T = 31.5 °C, pH = 6.7 for the induction phase). In Figure 1 an exemplary SEM picture of IBs including measurement of the size is given. With this size analysis technique, an IB size standard deviation could be determined with an uncertainty of about 10%. We think that this 10% value is based on biological divergence of the sample, rather than measurement error. These pictures were used to define IB size dependence of an individual cultivation (Figure 1b). At later induction times, decrease in size and increase in standard deviation indicated ongoing cell death measured via flow cytometry and sugar accumulation (compare to Ref. [22,38]).

**Figure 2:**
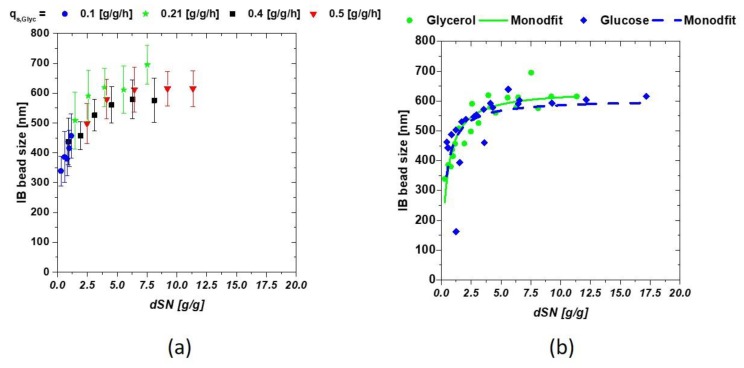
(**a**) IB-bead size dependencies on the amount of fed glycerol shown calculated as dSn value (**b**) dependence of the IB bead diameter when compared between glucose and glycerol.

We tested four different setpoints for q_s,C_ during the induction phase for both carbon sources and analyzed the resulting IB sizes. At high q_s,C_ early C-source accumulation could be observed with present product degradation. For easy comparison between different physiological feeding strategies and different used C-sources, the cumulative sugar uptake value, dSn [g/g], (Equation (2)) could be used making a normalization on the fed mass C-source in respect to the total biomass at the induction time [34]:

(2) $$dSn=\frac{\int_{0}^{t}m_{C}dt}{X\left(t_{0}\right)}$$

with m_C_ [g] being the fed mass of C-source, dt [h] the respective time interval and X(t_0_) [g] the total biomass at the start of induction. The induction time scale can now be exchanged for the dSn value during the induction phase. Low q_s,C_ values result in a low dSn value, while high q_s,C_ increases the values, as a high amount of C-source is fed throughout the process. The interaction between the IB bead size and dSn is presented in Figure 2a, comparing four runs with glycerol as C-source. Single bead size was strongly affected by the fed substrate starting with about 350 nm (threshold of the measurement principle) to a size over 600 nm in mean. A modified hyperbolic kinetic term could be used to describe this behavior of the mean diameters of the IBs in Equation (3):

(3) $$IB_{size}=IB_{size,max}*\frac{dSn}{K_{m}+dSn}$$

replacing the maximum reaction speed term of a Monod kinetic with the maximum IB size (IB_size,max_) and the substrate dependence through the respective dSn value. K_m_ defines the trigger point where half of the possible size is reached and may, therefore, be used as transition value for change of low sizes towards large IB-size interesting for further downstream applications. High feeding rates might help to reach this trigger point earlier, hence physiological limitations such as µ-decrease over induction time have to be considered, in order to prevent cell death. The results of the fit are given in Figure 2b for both C-sources. 

The previously mentioned hyperbolic fit can be seen by observing Figure 2b as IB-size is highly dependent on the amount of fed C-source. The performed fit yielded following constants for all cultivations at the constant parameters T and pH are given in Table 2. K_M_ represents the mass glycerol needed for 1 g of biomass to reach IB_size,max/2_.

Both C-sources showed very similar results using the fit given in Equation (3). This is actually surprising, as cellular uptake mechanism for glucose and glycerol are very different and are thought to influence production of recombinant proteins. IB bead size could not be influenced by the C-source (as differences seen here were within the standard deviation), but only by the respective amount of fed C-source (provided that T and pH are not altered during the induction phase). K_m_ and IB_size, max_ are highly product and induction dependent. Very small sizes were not easily accessible using SEM based techniques. Therefore, the highest error was to be expected in the approximation of the early induction times, as filter porosity was above the threshold. Maximum IB size was observed at approximately 620 nm for our model protein. IB_size, max_ therefore may reflect a physiological or physical limitation of IB growth inside *E. coli* cells.

### 3.2. Model-Based Approach for Prediction of IB Bead Size

**Figure 3:**
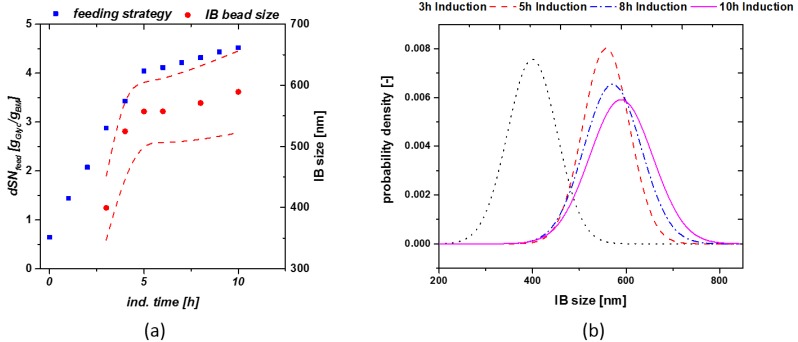
(**a**) Model cultivation with change in the q_s,C_ from 0.5 g/g/h to 0.1 g/g/h at a dSn value of about 4 g/g. The lower q_s,C_ value results in a high viable cell concentration at late induction times; (**b**) probability density plot of cultivations as a function of induction time.

These relations were the basis for the model workflow used for determination of size in real time during a process, based on the applied feeding strategy given in the discussion chapter. We applied the dSn principle for a cultivation in order to keep viable cell concentration high and receive a high product titer. The exact feeding strategy using the dSn value over induction time is given in Figure 3a (blue rectangles). A high q_s,C_ of glycerol was varied from 0.5 g/g/h to 0.1 g/g/h at a dSn value of 4 g/g. Corresponding size measurements are given in Figure 3a (red circles). As size and titer do show a linear correlation, the shift from a high to a q_s,C_ should result in optimal size at the time-point of harvest.

Exchanging a static feeding strategy with the feeding control shown in Figure 3a, does implement a size growth even at late induction times and is triggering maximum size towards the time-point of harvest. Optimized feeding strategies, varying µ over induction time, seem to boost the overall product titer [34]. The probability density plot shown in Figure 3b presents the size as a function of induction time for the given run. For establishment of the probability plot, we estimated a normal distribution of our IBs and used the standard deviation of our measurements using the SEM techniques. Within this cultivation only little deviation could be monitored towards end of induction time as q_s,C_ was adapted to 20% of the previous feeding rate. 

## 4. Discussion

Based on the q_s,C_ experiments with feed-forward approach, we derived empirical relation for IB size during our cultivation (compare to Figure 2b). For receiving IBs with defined size, low q_s,C_ values can be recommended, as protein aggregation is a very fast process, and therefore size alterations might be difficult to trigger. Very small defined sizes could be produced in the beginning and sizes are longer stable at these feeding rates. Altering induction strength with lactose showed similar dependence by using green fluorescent protein as model protein for mixed feed development [42]. Within this study, it was shown that IB-size is tunable by adjusting the lactose concentration as inducer. It was shown that low levels of lactose resulted in sizes only as big as 400 nm but using higher concentrations of lactose for induction, IB-bead sizes move up to 600 nm. However, this variation in size seems to be a result from insufficient induction, resulting from eventually given low lactose feeding rates. We want to highlight that inclusion body size is highly dependent on the amount of the fed C-source, given that the system is fully induced with IPTG. Based on these results, glycerol-fed cultivations resulted in identical size for IB beads compared to glucose, although glycerol does open up major advantages on a cultivation side. The derived straightforward dependence of the IB bead size based on hyperbolic saturation kinetics could be easily used in a model-based approach. Online application of the model during the process would help to reduce or even replace complex offline analytics.

Process control can generally be performed by applying mechanistic of empirical models for prediction of the performed process or for at least several key performance indicators (KPI) [43,44,45]. Regularly, the prediction of biomass and inhibiting metabolites are the quantities of interest and are tried to be simulated for predictive control of the bioprocess. Prediction of product attributes within the cultivation is challenging—especially in high dynamic microbial processes. For the development of our model, mechanistic links were established using the software Matlab 2017. Within this model only time of the induced fed batch was simulated, as no inclusion bodies were formed within batch and non-induced-fed batch (FB)-cultivation. Static inputs for the model consisted of volume [L], substrate in fermentation broth [g/L] and biomass [g/L] at timepoint t_induction_ = 0 h. Further feed concentration [g/L], Y_X/S_ [g/L] and q_s,max_ [g/g/h] were needed for modelling the behavior of the induction phase. Defining basic mass equations on fed-batch process behavior, prediction of classic process parameters like volume, substrate metabolization, biomass formation was established via the differential terms given beneath. Due to simplification, volume increase during fermentation was calculated via feed addition only:

$$x.S.dxdt={}^{\prime }\left(EQ.F_{in}/x\left(1\right)*p.c_{glu}.Value-x\left(2\right)\right)-EQ.qs*x\left(3\right){}^{\prime }\;x.DCW.dxdt={}^{\prime }x\left(3\right)*\left(EQ.qx-EQ.F_{in}*x\left(1\right)\right)\;Volume:\;x\left(1\right)=\left[L\right]\;Substrate:\;x\left(2\right)=\left[g\right]\;DCW:\;x\left(3\right)=\left[\frac{g}{L}\right]$$

**Figure 4:**
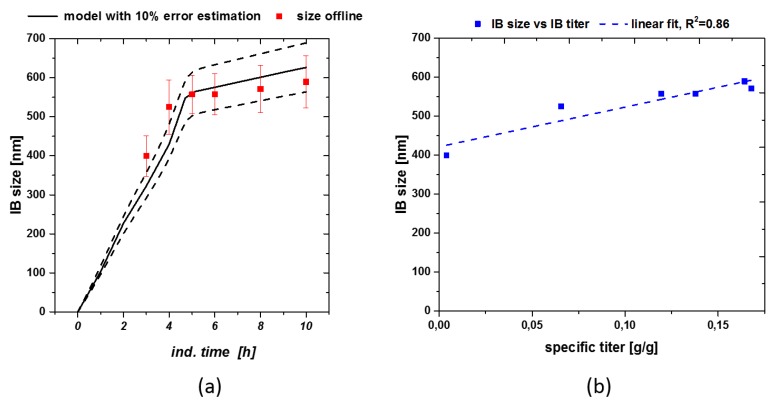
(**a**) Size modeling including real measured data with standard deviations. Especially early sizes could only be described using the model-based approach; (**b**) Size-titer correlation for fermentation with feeding strategy implied in Figure 3a.

With S being the substrate [g], F_in_ the feed [mL/h], c_Glu_ the feed concentration [g/L], DCW the dry cell weight [g/L], q_s_ the specific substrate feeding rate [g/g/h] – simulated in the model – and q_x_ the specific biomass generation rate [g/g/h] based on q_s_. This enabled us to simulate the process based on the inputs received from the static q_s,C_ experiments. Size could be easily modeled using the already presented hyperbolic saturation terms as helper equation in the model. Figure 4a shows the modeled size estimation based on the previously established kinetics in Equation (3). As acceptance criteria for product-based attributes we used 10% error of the model for size.

Size was well described by the model, resulting in only small variations, still lying within the standard deviation. Using the given model size estimation down to the beginning of the induction phase can be made, even if no analytical method is available for direct determination (e.g., SEM threshold). As size and titer show a linear correlation (compare to Figure 4b) overall product formation can be increased, applying the q_s,C_ shift from 0.5 to 0.1 g/g/h. Therefore, also titer could be estimated for a fixed process based on the presented model approach. Standard deviation of IB-bead size (plotted in Figure 3a also increases over time, which is in correlation with literature [42].

Model-based approaches for estimation of different feeding strategies can be easily used for adaption of control for IB based products. Deriving data from two to three fed-batch cultivations with different static q_s,C_ values is necessary to establish this model-based approach. Afterwards the optimized feeding strategy for the desired protein can be simulated and experimentally confirmed. This straightforward methodology makes this model-based approach a high beneficial tool for process development in the light of quality by design criteria in the industry.

## 5. Conclusions

In this work we aimed for analyzing the effect of varying the specific substrate feeding rate on IB CQA, such as IB size. Different cultivations could be easily compared using the previously established dSn value, as cumulated sugar uptake rate can be presented neatly arranged. Performing cultivations with altered feeding rates, showed, that maximum size was achieved at a dSn value of approximately 4 g/g. Feeding beneath a dSn value of 4 g/g resulted in highly time-resolved results, which might be useful for model development. As low feeding rates did not exceed the trigger-point in IB-formation (defined here as K_m_-value) these feeding strategies may not find an application in production scale, as IB titer is too low for further ongoing DSP applications, but are maybe interesting for other IB based products. Applying a q_s_-shift during induction phase boosted IB-formation even after dSn = 4 g/g was exceeded. Application of this feeding strategy kept cells in a viable state and further will help to achieve maximum size within shorter induction times. The relations for size based dSn approach could be used for process design but also for modelling of given product dependences.

We are currently investigating our model with other additional strains, trying to establish product-independent robust Upstream Processing. Triggering optimal IB-bead-size at the time-point of harvest will help to improve the cost-and time-intensive downstream processing.

## Figures and Tables

**Table 1:** Respective sugar concentrations in media composition.

Phase	Amount of C-source
Preculture	8 g/L
Batch-Media	20 g/L
Feed	either 300 g/L or 600 g/L

**Table 2:** Fitting results for hyperbolic-fit Equation (3) for IB bead size dependence.

Fit Parameters	Glucose	Glycerol
K_m_ [g/g]	0.33 +/− 0.14	0.42 +/− 0.06
IB_size,max_ [nm]	605.4 +/− 37	638 +/− 17

## Abbreviations

CDW	cell dry weight [g/L]
CQA	critical quality attribute
dSn	cumulative sugar uptake rate [g/g]
DSP	downstream processing
F_in_	Feed into the reactor [mL/h]
HPLC	high pressure liquid chromatography
IB	inclusion body
q_s,C_	specific substrate uptake rate [g/g/h]
SEM	scanning electron microscopy
T	temperature [°C]
